# Mango ginger (*Curcuma amada Roxb*.) may alleviate the effect of high‐fat diet/streptozotocin‐induced diabetes by activation of the GSK‐3β/Fyn/Nrf2 pathway

**DOI:** 10.1002/fsn3.3539

**Published:** 2023-07-04

**Authors:** Emrah Yazici, Emre Sahin, Nurhan Sahin, Mehmet Tuzcu, Kazim Sahin, Cemal Orhan

**Affiliations:** ^1^ Department of Animal Nutrition, Faculty of Veterinary Medicine Firat University Elazig Turkey; ^2^ Department of Animal Nutrition, Faculty of Veterinary Medicine Bingol University Bingol Turkey; ^3^ Department of Biology, Faculty of Science Firat University Elazig Turkey

**Keywords:** antioxidant capacity, diabetes, insulin resistance, mango ginger, Nrf2

## Abstract

Mango ginger (MG) exhibits antioxidant, anti‐inflammatory, and antihyperglycemic effects; however, the exact mechanism of action of MG extract in relation to its antidiabetic properties remains unclear. To investigate the potential antidiabetic effect of MG extract, we used a high‐fat diet (HFD)/low‐dose streptozotocin (STZ)‐induced type 2 diabetic rat model. A total of 28 male Wistar rats were randomly divided into four groups: (i) Control, (ii) MG (50 mg/kg/day of MG extract), (iii) HFD + STZ (40 mg/kg i.p.), and (iv) HFD + STZ + MG. Following a 12‐week administration of MG extract, significant reductions were observed in serum glucose, insulin, free fatty acid, cholesterol, and triglyceride levels in diabetic rats (*p* < .0001 for all). MG extract supplementation led to an increase in the total antioxidant capacity of the serum and a decrease in malondialdehyde (MDA) levels in both the serum and liver (*p* < .0001). Furthermore, hepatocellular fat accumulation was partially attenuated in the HFD + STZ + MG group. Notably, MG extract inhibited glycogen synthase kinase‐3β (GSK‐3β) in the liver (*p* < .01) and downregulated Fyn expression, resulting in elevated nuclear factor erythroid 2–related factor 2 (Nrf2) activity in the HFD + STZ + MG group compared to the HFD + STZ group (*p* < .05). The increased activity of Nrf2 in the HFD + STZ + MG group likely promoted the upregulation of heme oxygenase 1 (HO‐1) in the liver (*p* < .0001). In conclusion, MG extract may exert antidiabetic effects by augmenting the antioxidant defense system through the regulation of GSK‐3β/Fyn/Nrf2 in a rat model of type 2 diabetes.

## Graphical abstract

Type 2 diabetes is one of the main health problems worldwide and the GSK‐3β/Fyn/Nrf2 signaling pathway is a target for diabetes treatment. Mango ginger extract exhibits antidiabetic activity, reduces liver fat accumulation, and mediates the GSK‐3β/Fyn/Nrf2 signaling pathway.

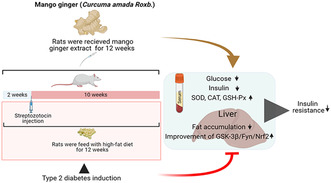

## 1 INTRODUCTION

Type 2 diabetes represents a multifaceted metabolic syndrome marked by disrupted regulation of carbohydrate, fat, and protein metabolism, resulting in increased levels of glucose and lipids in the blood. The interplay of metabolic abnormalities, chronic inflammation, and vascular dysfunction contributes to various complications associated with type 2 diabetes, including fatty liver, cardiovascular disease, retinopathy, and nephropathy (Rohm et al., 2022). Regrettably, the prevalence of diabetes is increasing significantly, and projections suggest that it may impact 783.2 million people worldwide by 2045 (Sun et al., 2022). Genetic factors are essential for diabetes, but unconscious and excessive consumption of high energy‐containing diets can trigger insulin resistance and hyperglycemia following obesity, eventually increasing susceptibility to type 2 diabetes (Magkos et al., 2020).

Accumulation of fat in the liver and pancreas may reduce insulin responsiveness, leading to insulin resistance and resulting in excessive glucose production in type 2 diabetes (Taylor et al., 2019). Elevating serum‐free fatty acids (FFAs) concentration during hepatic lipogenesis plays a pivotal role in developing insulin resistance observed in individuals with type 2 diabetes and obesity (Sobczak et al., 2019). The FFAs exert lipotoxic effects that contribute to hepatic damage by impairing insulin production in pancreatic β‐cells and exacerbating hepatic oxidative activity (Geng et al., 2021), mainly depending on reactive oxygen species (ROS) generation associated with hyperglycemia and insulin resistance (Kheiripour et al., 2019). Therefore, in type 2 diabetes, the antioxidant defense mechanism becomes vulnerable due to the attenuation of antioxidant enzyme activities, primarily in the liver (Singh et al., 2022).

In type 2 diabetes, improving the nuclear factor erythroid 2–related factor 2 (Nrf2)/kelch like ECH‐associated protein 1 (Keap1)/antioxidant response element signaling pathway mediates antioxidant defense, inhibits inflammation, and promotes cell survival; thus, it may attenuate oxidative damage (David et al., 2017). The activation and the nuclear accumulation of Fyn are partially stimulated by glycogen synthase kinase‐3β (GSK‐3β) (Zhang et al., 2012), which reduces insulin sensitivity and hepatic glycogen storage in type 2 diabetes (Gao et al., 2011). The GSK‐3/Fyn/Nrf2 signaling pathway enhances the antioxidant defense system in type 2 diabetic rats (Liu et al., 2018). In this pathway, glycogen synthase kinase‐3 (GSK‐3) and Fyn, a protein tyrosine kinase, are involved in the regulation of the nuclear factor erythroid 2‐related factor 2 (Nrf2) (Liu et al., 2018; Shang et al., 2015). Nrf2 is a transcription factor that plays a central role in activating the expression of various antioxidant enzymes and proteins (Bitar & Al‐Mulla, 2011; Wang et al., 2019). When this signaling pathway is activated, it leads to the phosphorylation and subsequent inhibition of GSK‐3 and Fyn. In turn, the inhibition of GSK‐3 and Fyn prevents the degradation of Nrf2 and allows it to translocate into the nucleus. Inside the nucleus, Nrf2 binds to antioxidant response elements (ARE) in the promoter regions of target genes, thereby initiating their transcription (Liu et al., 2018; Shang et al., 2015; Wang et al., 2019). Heme oxygenase‐1 (HO‐1), one of the target genes activated by this pathway, plays a vital role in breaking down heme groups, leading to the production of biliverdin, carbon monoxide, and free iron, all of which possess antioxidant effects and provide protection against oxidative stress (Bitar & Al‐Mulla, 2011; Yang & Wang, 2022). Previous studies indicate that natural phytochemical therapeutic agents could alleviate diabetic damage by regulating this signaling pathway (Liu et al., 2018; Shang et al., 2015; Sun et al., 2020).

*Curcuma amada Roxb*., commonly known as mango ginger (MG), is an important species recognized for its characteristic raw‐mango aroma and has a long history of traditional uses, encompassing both folk medicine and culinary preparations (Jatoi et al., 2007). It has many biological benefits, including anti‐inflammatory, antioxidant, antibacterial, anticancer antihyperglycemic, and antihypercholesterolemic activities (Erten et al., 2019; Mahadevi & Kavitha, 2020; Policegoudra, Aradhya, & Singh, 2011; Srinivasan et al., 2008). It has been reported that fresh and dried MG extracts contain upwards of 100 phytochemicals (Policegoudra, Aradhya, & Singh, 2011). The major chemical constituents of *C. amada* are myrcene, ocimene, ar‐turmerone, (Z)‐β‐farnasene, guaia‐6,9‐diene, cisβ‐ocimene, cis‐hydroocimene, transhydroocimene, α‐longipinene, α‐guaiene, linalool, β‐curcumene, and turmerone (Jatoi et al., 2007). Curcumin, a curcuminoid compound in the acetone extract of MG (Song et al., 2020), and caffeic acid, a major free phenolic acid of MG (Morroni et al., 2018), could reduce the GSK‐3β activity and improve the Nrf2 signaling. Furthermore, myrcene, a volatile component of MG, can potently inhibit inflammatory reactions and regulate serum glucose and insulin levels in diabetic rats (Yang & Liao, 2021).

Sarkar et al. (2019) assumed that the antihyperglycemic effect of MG extract might stem from the stimulation of pancreatic β cells for insulin secretion in streptozotocin (STZ)‐induced diabetic rats. Similarly, Azhar et al. (2019) indicated that the antidiabetic effect of ethanolic mango seed extract was related to insulin production stimulation in the pancreas. However, the molecular action mechanism of MG in type 2 diabetes needs to be clarified. Therefore, we investigated how MG affects the GSK‐3/Fyn/Nrf2 pathway, a crucial regulator of the antioxidant defense system in type 2 diabetes, using a rat model. We aimed to unravel the specific impact of MG on this pathway, thereby enhancing our understanding of its role in modulating the antioxidant defense system in diabetic conditions.

## 2 MATERIALS AND METHODS

### 2.1 Animals

A power analysis was conducted using the G*Power (Version 3.1.9.4) software to determine the required total sample size. We calculated a sample size of 28 rats with a 0.05 type I error, 0.7 effect size, and 80% power. The male Wistar Albino rats (age: 8 weeks, body weight: 170 ± 20 g) were purchased from the Experimental Research Unit of Firat University and housed in propylene cages (43 × 27 × 15 cm) with free access to food and water at standard conditions (22 ± 2°C, humidity, 55% ± 5%). The Animal Experiments Local Ethics Committee of Firat University approved the study following Directive 2010/63/EU, and the study was reported according to Animal Research: Reporting of In Vivo Experiments principles (protocol number: 2017/89‐169).

### 2.2 Experimental design

After 1 week adaptation period, 28 rats were separated into four groups (*n* = 7) as follows: (i) Control (fed with a standard diet), (ii) MG (fed with a standard diet, and 50 mg/kg/day MG extract was administered orally), (iii) High‐fat diet (HFD) + STZ (fed with a HFD, and 40 mg/kg STZ was administered intraperitoneally), and (iv) HFD + STZ + MG (fed with a HFD, and STZ and MG administered). The composition of the standard diet and HFD is given in Table 1.

**TABLE 1:** Ingredients of standard and high‐fat diet (HFD) fed to rats.

Ingredients, %	Standard diet	HFD
Casein	20.00	20.00
Corn Starch	57.95	15.00
Sucrose	5.00	14.95
Vegetable oil	7.00	–
Beef tallow	–	40.00
Cellulose	5.00	5.00
Mineral premix^a^	3.50	3.50
Vitamin premix^b^	1.00	1.00
L‐cysteine	0.30	0.30
Choline bitartrate	0.25	0.25

^a^ Modified AIN‐93G‐MX.

^b^ AIN‐93‐VX (No: 310025).

MG extract was obtained from OmniActive Health Technologies (Thane, India). The MG and HFD + STZ + MG groups received 50 mg/kg/day of MG extract (Sahin et al., 2017) via oral gavage throughout the study (12 weeks). A total of 14 animals were used for the type 2 diabetes model induced by a 40 mg/kg STZ (i.p., dissolved in 0.1 M citrate buffer) following 2 weeks of HFD feeding. After 72 h, blood samples were taken from the tail vein, and a glucometer (AccuChek Active; Roche Diagnostics) was used to measure fasting blood glucose levels. The rats with over 250 mg/dL (13.9 mmol/L) of blood glucose were accepted as diabetic and randomly divided into two groups. HFD feeding was continued for 10 weeks after diabetes induction (Sahin et al., 2019).

At the end of the experiment, animals were fasted overnight (12 h) and anesthetized using xylazine/ketamine hydrochloride; then, they were sacrificed by cervical dislocation. Following centrifugation of the blood (3000 rpm for 10 min), serum samples were obtained. The livers were removed immediately and stored at −80°C. A part of the liver samples from each animal was transferred into a 10% buffered formalin solution.

### 2.3 Biochemical analyses

The level of serum alanine transaminase (ALT), aspartate transaminase (AST) activities and the concentrations of glucose, cholesterol, triglyceride, creatinine, and blood urea nitrogen (BUN) were determined by a biochemistry analyzer (Samsung LABGEO^PT10^, Suwon, Korea) (Erten et al., 2020). Serum FFA level was measured with a diagnostic kit (Boehringer Mannheim, Germany), according to the colorimetric method (Shimizu et al., 1980).

Serum insulin levels were detected by Sandwich‐Enzyme‐Linked Immunosorbent Assay (ELISA) kit (Elabscience, Wuhan, China) with the help of a microplate reader (Elx‐800; Bio‐Tek Instruments Inc.). Serum total antioxidant capacity (TAC) was detected by a kit based on the ABTS reduction method (Rel Assay Diagnostics, Gaziantep, Turkey) (Erel, 2004). The inter‐ and intra‐assay coefficients were 4.18% and 4.20% for insulin and 3.3% and 2.8% for TAC, respectively.

MDA levels in the serum and liver were determined using high‐performance liquid chromatography (HPLC, Shimadzu, Tokyo, Japan), as previously described by Sahin et al. (2019). Briefly, the serum samples and liver homogenates were prepared for loading to the autosampler (SIL‐20A). 30 mM KH_2_PO_4_–methanol (82.5 + 17.5, v/v%, pH 3.6) mixture was used as a mobile phase in the HPLC, equipped with a CTO‐10 AS VP column, LC‐20 AD pump, and UV–VIS SPD‐10 AVP detector. The injection volume was 20 μL for each sample.

### 2.4 Insulin homeostasis

The homeostasis model assessment of insulin resistance (HOMA‐IR) was detected, as stated by Uckun et al. (2022). The average fasting blood glucose levels (4.38 mmol/L) and insulin (10.63 mIU/L) were detected, and the coefficient was computed as 46.52. The following formula was used to determine HOMA‐IR:

$$\text{HOMA}-\mathrm{IR}=\left[\text{fasting glucose}\;\left(\text{mmol}/L\right)\right]\times \left[\text{fasting insulin}\;\left(\mathrm{mlU}/L\right)\right]/46.52$$

In this study, we calculated the cut‐off value as 3.25 by receiver operating characteristic analysis, and rats presenting HOMA‐IR ≥3.25 were accepted as insulin resistant.

For evaluating the β‐cell function and insulin sensitivity, we calculated HOMA‐β and quantitative insulin sensitivity check index (QUICKI) with the following formulas (Wickramasinghe et al., 2022):

$$\text{HOMA}-\beta =\left[20\times \text{fasting insulin}\;\left(\mathrm{mlU}/L\right)\right]/\left[\text{fasting glucose}\;\left(\text{mmol}/L\right)-3.5\right]$$

$$\text{QUICKI}=1/\left[\mathrm{Log}\;\text{fasting insulin}\;\left(\mathrm{mlU}/L\right)+\mathrm{Log}\;\text{fasting glucose}\;\left(\mathrm{mg}/\mathrm{dL}\right)\right]$$

### 2.5 Histopathological analysis

After being removed from the formalin solution, the liver samples were embedded in paraffin blocks. The paraffin blocks were sliced into 4‐m‐thick sections using a microtome and subsequently mounted onto slides. The slides were visualized by the usual hematoxylin–eosin staining procedure and evaluated under a light microscope (Olympus Co.) by an expert.

### 2.6 Western blot analysis

The liver samples of each group were pooled and homogenized with ice‐cold radioimmunoprecipitation assay buffer containing a protease inhibitor. The protein amount of the homogenates was detected using a NanoDrop (Thermo Fisher Scientific, Inc.). The homogenates were mixed with 2× Laemmle buffer and boiled for 5 min. Next, samples were separated by 12% sodium dodecyl sulfate‐polyacrylamide gel electrophoresis for 2 h. The gels were placed in a semi‐dry blotter, and proteins were transferred into the nitrocellulose membrane. We used a 5% bovine serum albumin solution to prevent non‐specific protein binding to block the nitrocellulose membranes. After, rat‐specific primary antibodies (GSK‐3β, Fyn, Nrf2, Keap‐1, and HO‐1, Santa Cruz Biotechnology, CA, USA) were incubated with the nitrocellulose membranes overnight at 4°C. Next, these membranes were incubated with HRP‐conjugated secondary antibodies for 2 h. β‐Actin (Santa Cruz Biotechnology, CA, USA) was used to confirm equal protein loading. The protein bands were visualized by the diaminobenzidine substrate method, and the density of the bands was measured using the Image J program (National Institute of Health, Bethesda, USA) on a computer.

### 2.7 Statistical analyses

Statistical analyses were performed in SPSS software 22.0 (IBM Corp.). Data were shown as the mean and standard deviation. The multiple comparisons between the groups were carried out by one‐way ANOVA and Tukey's or Tamhane's T2 post hoc test. *p* < .05 level indicates the difference between groups.

## 3 RESULTS

### 3.1 Body weight and serum biochemical parameters

**FIGURE 1:**
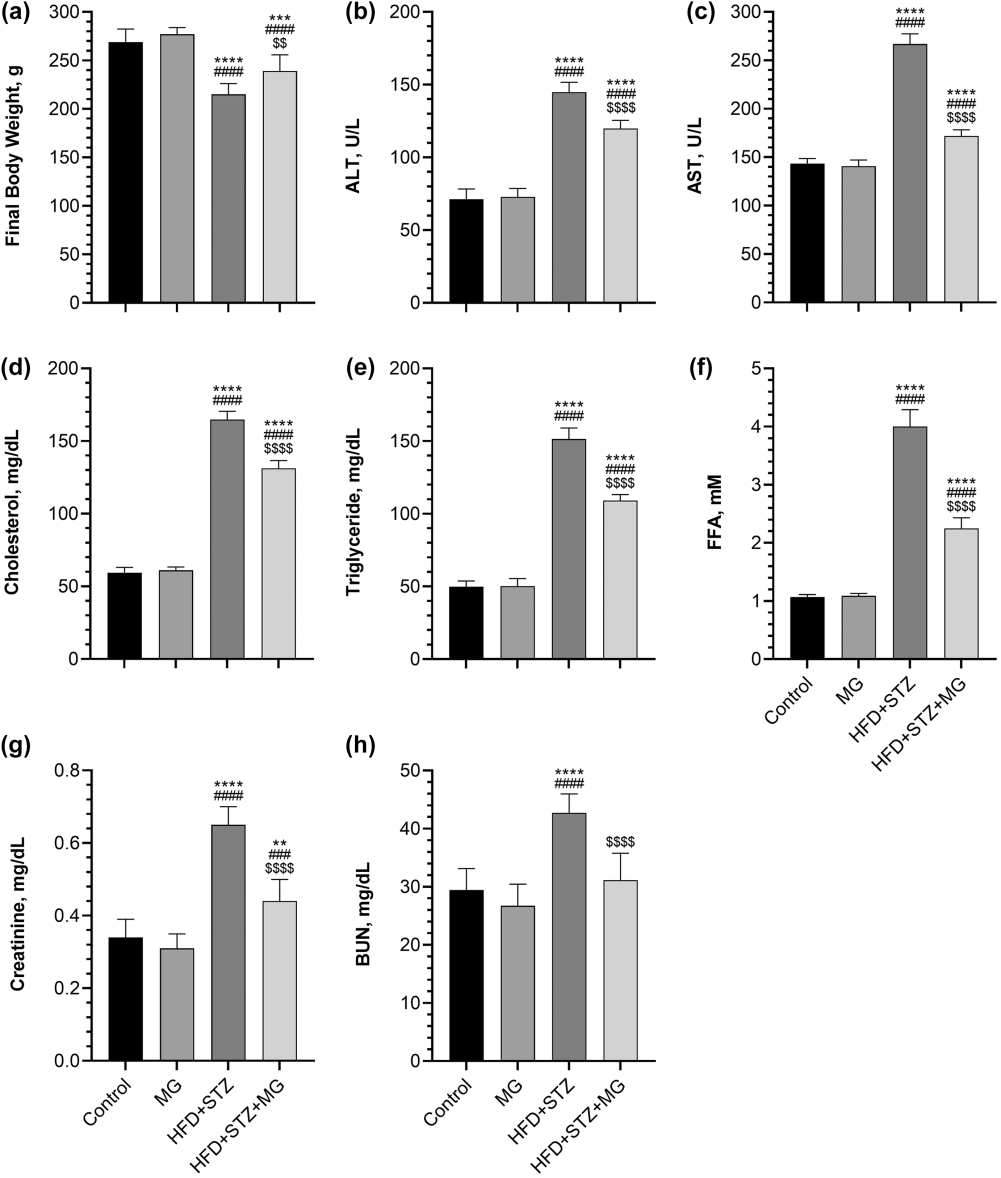
Effects of mango ginger (MG) supplementation on body weight (a), alanine transaminase (ALT, b), aspartate transaminase (AST, c), cholesterol (d), triglyceride (e), free fatty acid (FFA, f), creatinine (g), and blood urea nitrogen (BUN, h) in high‐fat diet (HFD)/streptozotocin (STZ)‐induced type 2 diabetic rats. The error lines on the bars point out the standard deviation of the mean. Different symbols above the bars indicate statistical differences among the groups (ANOVA and Tukey's *post‐hoc* test; **p < .01 and ****p < .0001 compared to the control group; ^###^ *p* < .001 and ^####^ *p* < .0001 compared to the MG group; ^$$^ *p* < .01 and *p* < .0001 compared to the HFD + STZ group).

As seen in Figure 1, MG supplementation did not significantly alter body weight (Figure 1a) in healthy rats (*p* > .05). HFD/STZ‐induced type 2 diabetes reduced body weight compared to healthy control rats, and MG supplementation partially prevented this reduction (*p* < .01). Serum ALT (+203%), AST (+186%), cholesterol (+277%), triglyceride (+304%), FFA (+374%), creatinine (+191%), and BUN (+145%) levels increased in the HFD + STZ group compared to the control group (Figure 1b–h, *p* < .0001). Although MG supplementation significantly decreased serum ALT (−17%), AST(−36%), cholesterol (−20%), triglyceride (−28%), FFA (−44%), creatinine (−33%), and BUN (−27%) levels in the HFD + STZ + MG group compared to the HFD + STZ group (*p* < .0001), the level of these parameters was still higher than in the control and MG groups (*p* < .01 for creatinine, *p* < .0001 for others), except BUN (*p* > .05).

### 3.2 Glucose, insulin, HOMA‐IR, HOMA‐β, and QUICKI

**FIGURE 2:**
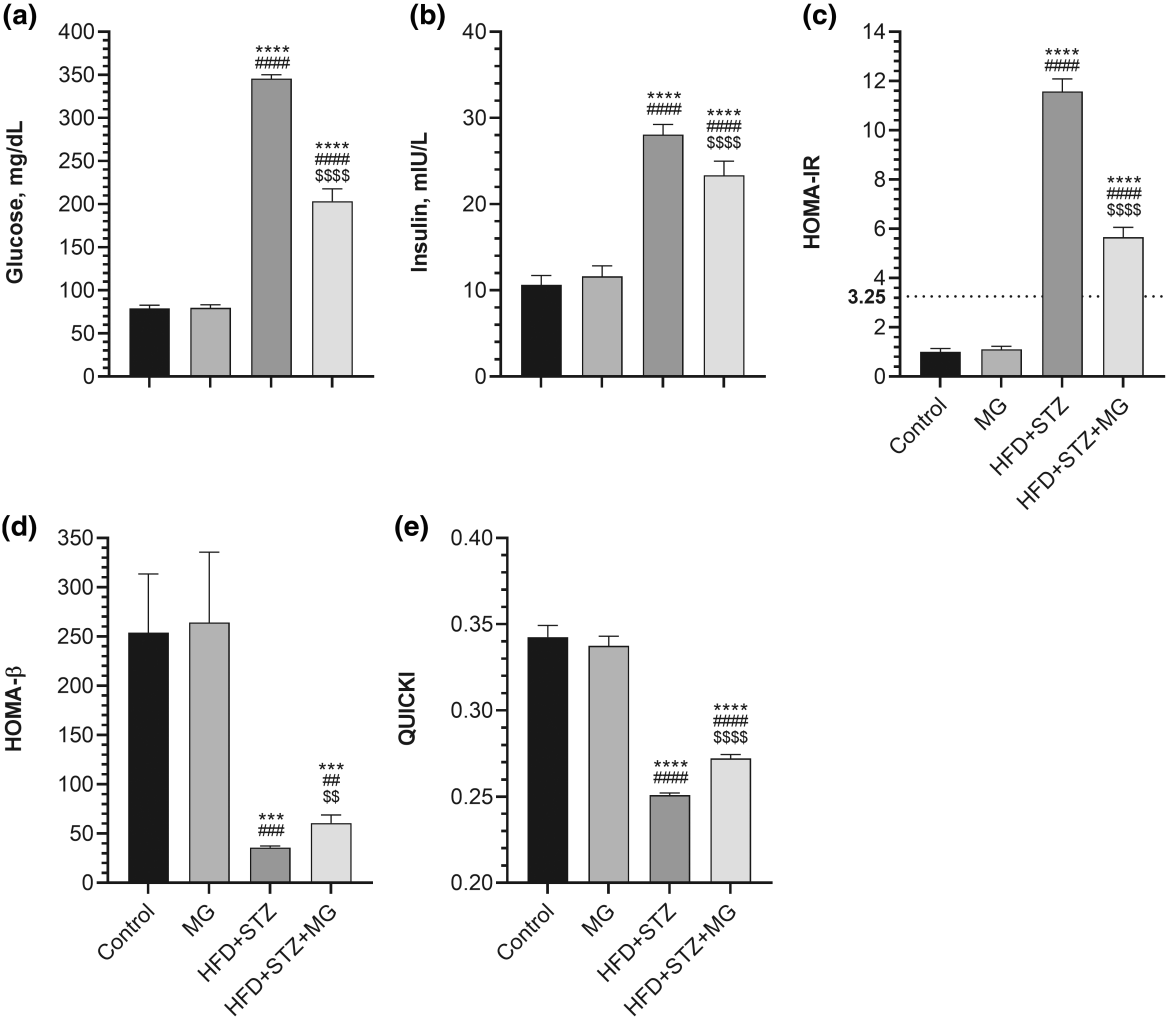
Effects of mango ginger (MG) supplementation on serum glucose (a), serum insulin (b), homeostasis model assessment of insulin resistance (HOMA‐IR, c), the β‐cell function (HOMA‐β, d), and quantitative insulin sensitivity check index (QUICKI, e) in high‐fat diet (HFD)/streptozotocin (STZ)‐induced type 2 diabetic rats. The error lines on the bars points out the standard deviation of the mean. Different symbols above the bars indicate statistical differences among the groups [ANOVA and Tukey's (for body weight, serum glucose, serum insulin) or Tamhane's T2 (for HOMA‐IR, HOMA‐β, and QUICKI) *post‐hoc test*; ***p < .001 and ****p < .0001 compared to the control group; ^##^ *p* < .01, ^###^ *p* < .001, and ^####^ *p* < .0001 compared to the MG group; ^$^ *p* < .05; ^$$^ *p* < .01 and ^$$$$^ *p* < .0001 compared to the HFD + STZ group].

In the HFD + STZ group, serum glucose and insulin levels exhibited a substantial increase of 438% and 264%, respectively, compared to the control group (Figure 2a,b, *p* < .0001, for all). There was a reduction in serum glucose and insulin levels in MG‐supplemented diabetic rats compared to non‐supplemented diabetic rats (*p* < .0001 for all). However, MG did not diminish the serum glucose and insulin to the control group level (*p* < .0001). We found that HOMA‐IR, a calculation that indicates insulin resistance, was higher in the HFD + STZ group than in the control and HFD + STZ + MG groups (Figure 2c, *p* < .0001). None of the diabetic rats receiving MG supplementation exhibited a HOMA‐IR value lower than the defined cut‐off value of 3.25. Figure 2d,e shows that the β‐cell function (HOMA‐β) and insulin sensitivity (QUICKI) were suppressed in diabetic rats. MG supplementation markedly relieved this suppression in diabetic rats compared to non‐supplemented diabetic rats (*p* < .01 for HOMA‐β, *p* < .0001 for QUICKI).

### 3.3 MDA and TAC level

**FIGURE 3:**
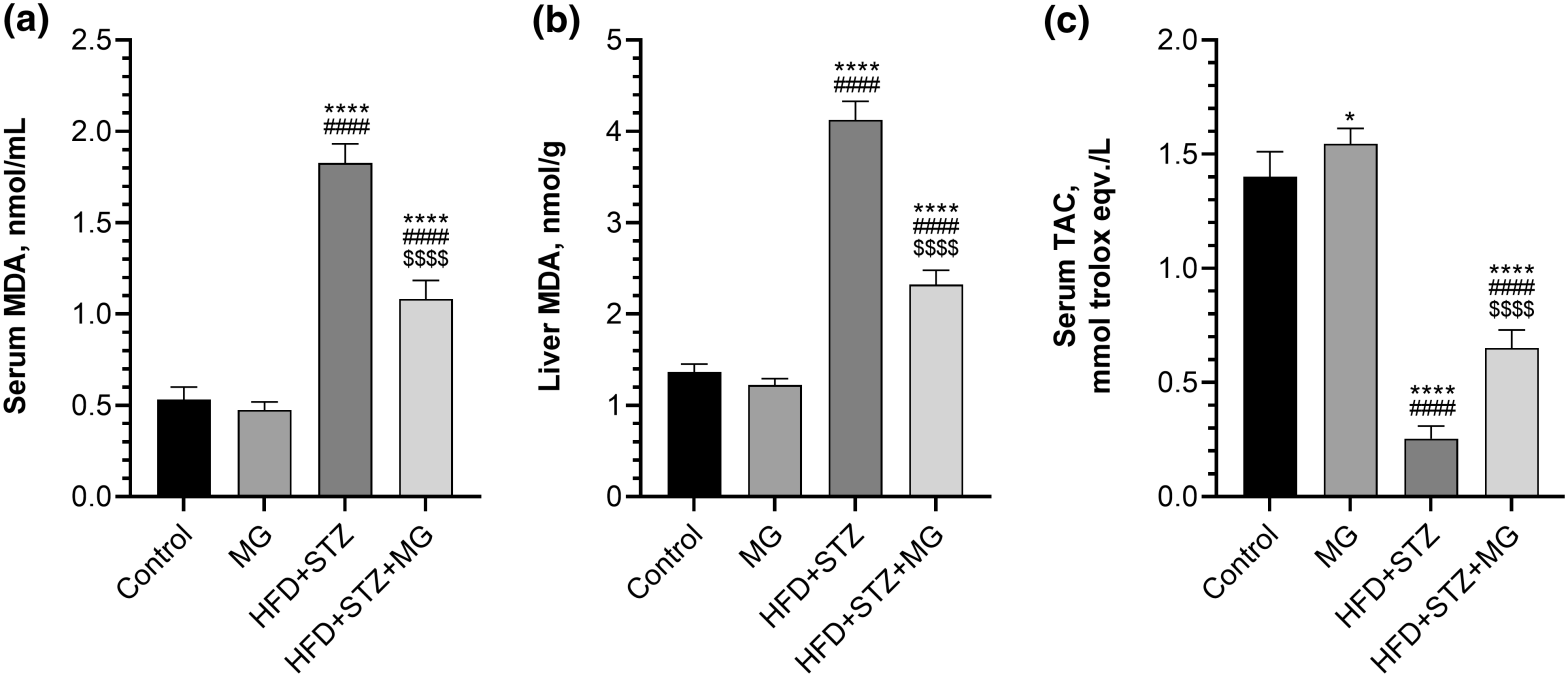
Effects of mango ginger (MG) supplementation on serum malondialdehyde (MDA, a), liver MDA (b), and total antioxidant capacity (TAC, c) in high‐fat diet (HFD)/streptozotocin (STZ)‐induced type 2 diabetic rats. The error lines on the bars points out the standard deviation of the mean. Different symbols above the bars indicate statistical differences among the groups (ANOVA and Tukey's *post‐hoc test*; *p < .05 and ****p < .0001 compared to the control group; ^####^ *p* < .0001 compared to the MG group; ^$$$$^ *p* < .0001 compared to the HFD + STZ group).

As expected, the induction of diabetes resulted in elevated levels of serum MDA (+344%) and liver MDA (+302%) compared to the control group (Figure 3a,b, *p* < .0001 for all). MG supplementation decreased the serum and liver MDA concentration in the HFD + STZ + MG group compared to the HFD + STZ group (*p* < .0001 for all). In contrast, the TAC level (−81%) was decreased in the HFD + STZ group compared to the control group (Figure 3c, *p* < .0001). However, the HFD + STZ + MG group has higher TAC levels than the HFD + STZ group (*p* < .0001), TAC levels in MG‐supplemented diabetic rats were still lower than in healthy control rats (*p* < .0001). Additionally, MG supplementation increased serum TAC levels in the MG group compared to the control group (*p* < .05).

### 3.4 GSK‐3β, Fyn, Nrf2, Keap‐1, and HO‐1 protein level

**FIGURE 4:**
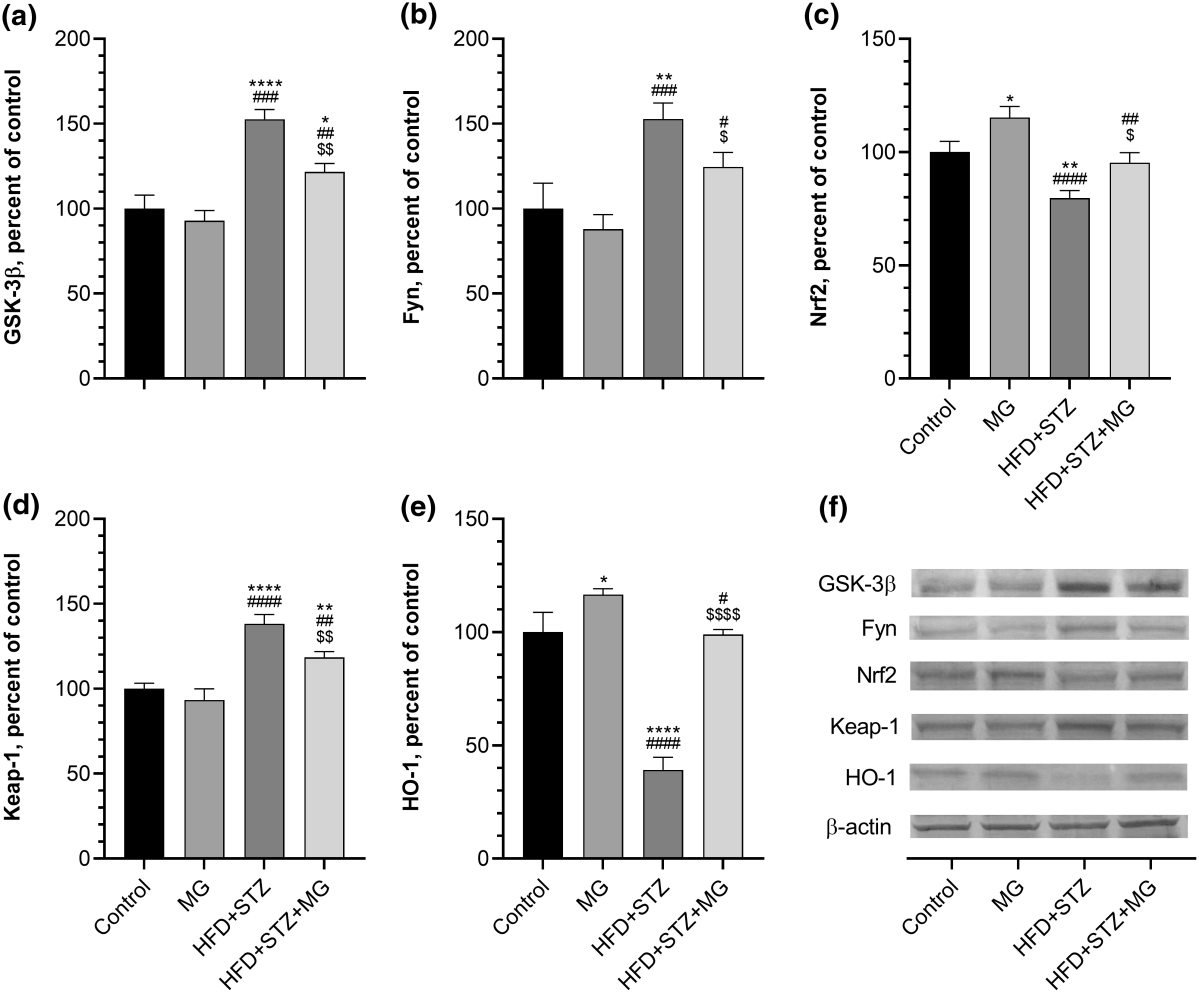
Effects of mango ginger (MG) supplementation on liver GSK‐3β (a), Fyn (b), Nrf2 (c), Keap‐1 (d), and HO‐1 (e) in high‐fat diet (HFD)/streptozotocin (STZ)‐induced type 2 diabetic rats. β‐Actin was referenced to ensure equal protein loading. Blots were repeated at least three times (*n* = 3) and representative blots are shown (f). Data are demonstrated as a percent of the control value. The error lines on the bars points out the standard deviation of the mean. Different symbols above the lines indicate statistical differences among the groups (ANOVA and Tukey's *post‐hoc test*; *p < .05, **p < .01, and ****p < .0001 compared to the control group; ^#^ *p* < .05, ^##^ *p* < .01, ^###^ *p* < .001, and ^####^ *p* < .0001 compared to the MG group; ^$^ *p* < .05, ^$$^ *p* < .01, and ^$$$$^ *p* < .0001 compared to the HFD + STZ group).

We determined liver GSK‐3β, Fyn, Nrf2, Keap‐1, and HO‐1 levels to explore the molecular action mechanism of MG on antioxidant status and antidiabetic effect. Our study revealed a significant relative increase in liver GSK‐3β (Figure 4a, *p* < .0001), Fyn (Figure 4b, *p* < .01), and Keap‐1 (Figure 4d, *p* < .0001) levels by 53%, 53%, and 38%, respectively, in response to the induction of type 2 diabetes compared to the levels observed in healthy control rats. The activity of GSK‐3β (*p* < .01), Fyn (*p* < .05), and Keap‐1 (*p* < .01) was significantly attenuated by MG in the HFD + STZ + MG group compared to the HFD + STZ group. Interestingly, liver Fyn level was not statistically different between the HFD + STZ + MG and the control groups (*p* > .05). In contrast to GSK‐3β, Fyn, and Keap‐1, liver Nrf2 (Figure 4c, *p* < .01) and HO‐1 (Figure 4e, *p* < .0001) levels were lowered by 20% and 60%, respectively, in the HFD + STZ group compared to the control group. Liver Nrf2 and HO‐1 levels were increased by MG supplementation in diabetic rats, and no difference was found between HFD + STZ + MG and control groups (*p* > .05). Moreover, as parallel in serum TAC levels, MG supplementation increased Nrf2 and HO‐1 activity in the MG group compared to the control group (*p* < .05).

### 3.5 Histopathology

**FIGURE 5:**
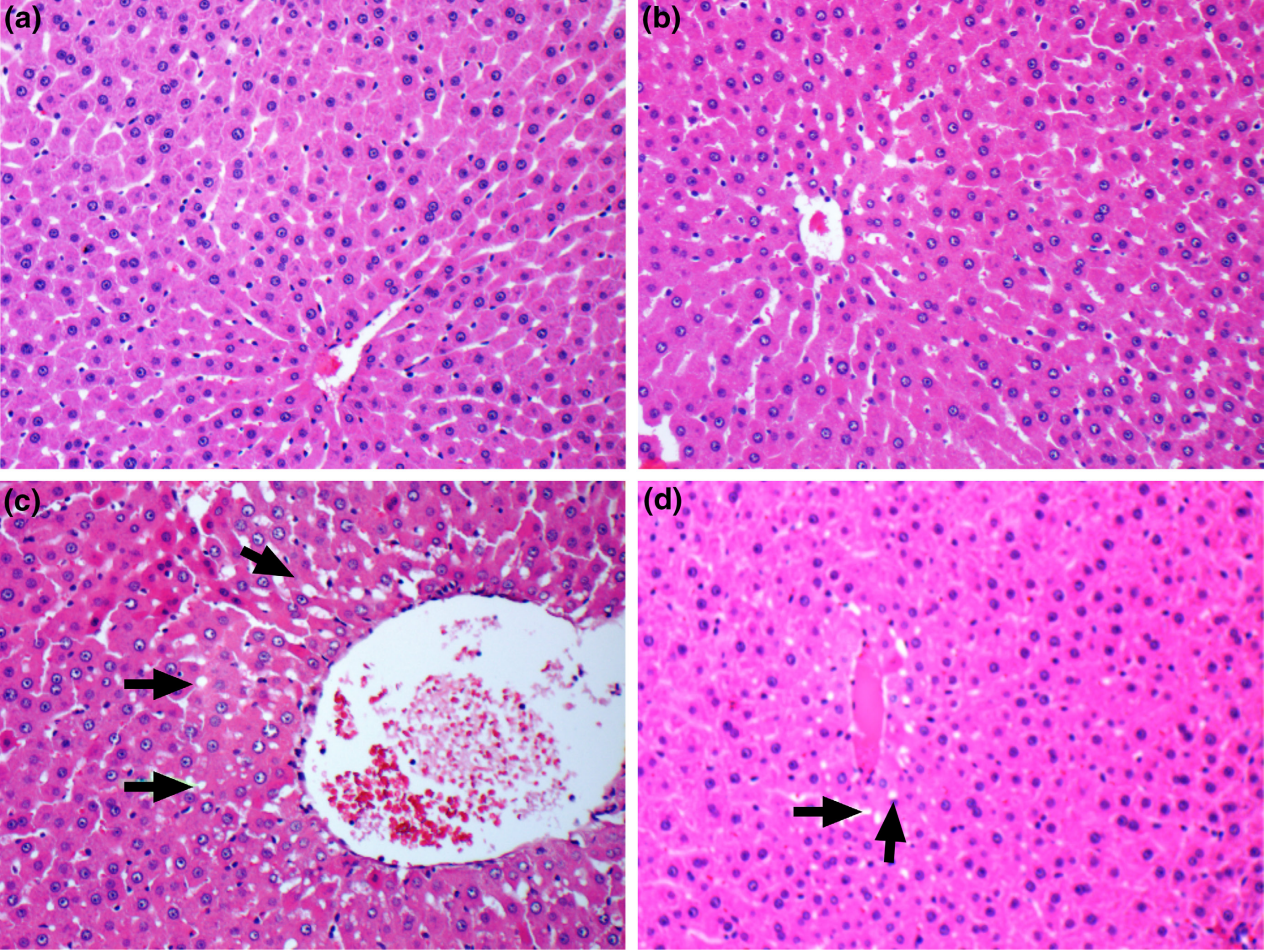
Effects of mango ginger (MG) supplementation on histological alterations in the liver in high‐fat diet (HFD)/streptozotocin (STZ)‐induced type 2 diabetic rats (H&E, 200X). HFD/STZ‐induced rats had fat inclusions within hepatocytes (black arrows). (a) Control; (b) MG; (c) HFD + STZ and (d) HFD + STZ + MG.

The liver tissue had a normal histological structure without fibrosis and fat accumulation in the healthy control and MG‐supplemented group (Figure 5a,b). We observed a normal central vein and polygonal hepatocytes with well‐defined cell borders. In diabetic rats, hepatocytes displayed an irregular shape and lacked proper polygonal organization. Additionally, hepatocellular degeneration and fatty inclusions were detected in hepatocytes (Figure 5c). We observed that MG supplementation partly reversed degenerative alteration in hepatocytes and reduced fatty acid accumulation in the HFD + STZ + MG group compared to the HFD + STZ group (Figure 5d).

## 4 DISCUSSION

The combination of HFD and low‐dose STZ is widely used to establish the type 2 diabetes model in rats (Sahin et al., 2019; Uckun et al., 2022; Wickramasinghe et al., 2022). Excessive fat intake elevated serum and liver FFA may promote inflammatory hepatic damage and insulin resistance (Ma et al., 2018), leading to the deterioration of glucose metabolism and subsequent hyperglycemia, hyperinsulinemia, and dyslipidemia (Sahin et al., 2019). Also, STZ targets the insulin‐producing the β‐cells and may break down tissue proteins, resulting in body weight loss (Erten et al., 2020). Therefore, this study confirmed that with body weight loss, serum triglyceride, cholesterol, FFA, insulin, and glucose levels were tightly associated with type 2 diabetes and significantly increased in HFD/STZ rats.

Additionally, as seen in the histopathological examination, type 2 diabetes likely induced hepatic damage depending on lipotoxicity, and serum ALT and AST levels were markedly raised in our diabetic groups, as Goboza et al. (2019) reported. The diabetic rats also exhibited higher creatinine and BUN levels than the control rats, likely due to the nephropathic effect of hyperglycemia (Kaikini et al., 2020). These results show that we successfully mimicked type 2 diabetes with HFD and low‐dose STZ injection in Wistar albino rats.

The biological activity of MG stems from its phytochemical composition involving curcuminoids, phenolic acids, terpenoids (Policegoudra, Aradhya, & Singh, 2011), and flavonoids (Prema et al., 2014), as in plenty of medicinal plants used against diabetes (Paramanick & Sharma, 2017). Methanol extracts of MG have higher phenolic content that correlates with antioxidant activity (Policegoudra, Chandrashekhar, et al., 2011) and antidiabetic potential (Dias et al., 2017). Syiem et al. (2011) reported that the methanol extract of MG exhibits a metformin‐like antihyperglycemic and hypoglycemic effect in alloxan‐induced diabetic mice. This antihyperglycemic activity probably originated from improved hexokinase activity that mediates glucose utilization (Sarkar et al., 2019). Besides enhanced hexokinase activity, aqueous methanol extract of MG may show the β‐cell regenerating and insulin‐normalizing effects in diabetic rats (Mitra et al., 2020). We found that HFD/STZ rats were highly insulin resistant (HOMA‐IR) and had reduced β‐cell functions (HOMA‐β) and insulin sensitivity index (QUICKI). However, MG extract regulated insulin activity, possibly by increasing glucose utilization and restoring pancreatic β‐cells function.

The present study supported that MG improves liver function and lipid metabolism in diabetes. According to Srinivasan et al. (2008), both whole MG and curcumin‐containing portions of MG can reduce cholesterol in the liver and serum of high cholesterol‐fed rats. Similarly, it was reported that the curcumin‐free MG extract was capable of reversing hyperlipidemia by elevating lipoprotein lipase activity in triton WR‐1339 induced‐hyperlipidemic rats (Srinivasan & Chandrasekhara, 1993). In diabetic rats, the aqueous methanol extract of MG, which contains phenols, alkaloids, and flavonoids, has been shown to alleviate impaired liver function owing to its antioxidant properties and, as demonstrated in this study, reduce serum ALT and AST levels (Mitra et al., 2020). Moreover, the lipid‐lowering effect of MG in both serum and liver (Srinivasan et al., 2008) probably contributed to the suppression of FFA‐originated insulin resistance in peripheral tissues and increased insulin sensitivity in HFD/STZ rats.

Previously many studies have reported that antioxidant capacity was reduced by type 2 diabetes (Erten et al., 2020; Goboza et al., 2019; Kheiripour et al., 2019; Sahin et al., 2019). Also, it is well‐known that MDA, a lipid peroxidation marker, levels significantly increase in serum (Sahin et al., 2019) and liver (Kheiripour et al., 2019) due to lipotoxicity, low‐grade inflammation, and oxidative stress in HFD/STZ‐induced type 2 diabetes (Singh et al., 2022). MG extract exhibits antioxidant effects by upregulating antioxidant enzymes (Mitra et al., 2020; Sarkar et al., 2019) and inhibiting inflammatory cytokines (Karataş et al., 2020). This study determined that the administration of 50 mg/kg BW/day MG for 12 weeks in type 2 diabetic rats resulted in an improvement in TAC and a decrease in MDA levels. In line with the current findings, Sahin et al. (2017) reported that the administration of 50 mg/kg of MG for 8 weeks reduced muscle MDA levels, concomitant with an elevation in antioxidant enzyme levels (superoxide dismutase and glutathione peroxidase) in exercise‐trained rats. Phenolic compounds can scavenge free radicals and efficiently mitigate tissue damage caused by ROS (Zeb, 2020). Moreover, some terpenoid bioactive compounds (amadannulen and amadaldehyde) isolated from MG exhibit lipid peroxidation activity (Policegoudra, Aradhya, & Singh, 2011). Thus, the antioxidant effect of MG can be attributed to the presence of phenolic compounds, mainly such as caffeic acid, gentisic acid, ferulic acid, gallic acid (Kiokias et al., 2020), as well as curcuminoids (Llano et al., 2019), and terpenoids (Policegoudra, Aradhya, & Singh, 2011).

In agreement with the present results, a study conducted by Sun et al. (2020) has demonstrated that HFD/STZ‐induced type 2 diabetes could increase the activity of GSK‐3β, resulting in enhanced accumulation of Fyn in the cell nucleus (Yi et al., 2018). Fyn promotes the nuclear export of Nrf2, leading to its degradation (Yi et al., 2018). Increasing the ratio of Nrf2 to Keap1 may enhance the Nrf2/Keap1 signaling activity and prevent Nrf2 degradation (Alves et al., 2019; Zhang et al., 2020). Moreover, GSK‐3β may suppress the Nrf2/HO‐1 pathway in diabetic rats and negatively affects antioxidant signaling (Shen et al., 2017). As demonstrated in an earlier type 2 diabetic wound model (Bitar & Al‐Mulla, 2011), the current results indicated that HFD/STZ‐induced type 2 diabetes leads to irregular Nrf2/Keap1 and Nrf2/HO‐1 signaling by unbalancing GSK‐3β/Fyn/Nrf2 signaling in the liver.

Many phytochemical therapeutics may relieve diabetic damage by targeting the GSK‐3β/Fyn/Nrf2 signaling pathway (Liu et al., 2018; Shang et al., 2015; Sun et al., 2020; Zhang et al., 2012). However, since the effect of MG on GSK‐3β/Fyn/Nrf2 signaling is unclear, we tried to elucidate this effect partially. Previously Sahin et al. (2017) reported that MG extract boosts Nrf2 and HO‐1 levels and improves the antioxidant capacity of the aorta and muscle tissue in treadmill‐running rats (Sahin et al., 2017). Additionally, many studies declared that different phytochemical agents such as curcumin (Song et al., 2020) and caffeic acid (Morroni et al., 2018) found in MG could reduce GSK‐3β phosphorylation and stimulate Nrf2/HO‐1 signaling (Morroni et al., 2018; Song et al., 2020). Also, it has been reported that caffeic acid can directly inhibit Fyn activation in skin cells (Kang et al., 2009). Recently Razliqi et al. (2023) demonstrated that gentisic acid improves serum TAC by upregulating pancreatic Nrf2 signaling in type 2 diabetic mice. Ferulic acid can inhibit methotrexate‐induced hepatotoxicity by stimulating the Nrf2/HO‐1 signaling pathway (Mahmoud et al., 2020). These reports support our findings, which show that MG extract effectively inhibited GSK‐3β and Fyn activation while increasing Nrf2 and HO‐1 levels in the livers of type 2 diabetic rats. This beneficial effect is probably attributable to the abundant phenolic content of MG. Therefore, it could be concluded that MG shows Nrf2‐dependent antioxidant activity in diabetic rats.

One notable limitation of the present study is the absence of an analysis of the phytochemical composition of the MG. Therefore, specific antidiabetic effects of the particular chemical constituents and bioactive compounds in the MG remain unidentified and unexplored. Moreover, another limitation of this study is that no antidiabetic drugs (such as metformin) were used. Also, antioxidant enzymes such as hepatic superoxide dismutase and catalase were not measured.

## 5 CONCLUSION

This study showed for the first time that MG extract exhibits its antioxidant activity by regulating GSK‐3β/Fyn/Nrf2 signaling in the liver in type 2 diabetic rats. The Nrf2‐dependent antioxidant function of MG extract may have helped to reduce serum glucose, FFA, and insulin resistance and improve antioxidant capacity in type 2 diabetic rats. These promising results may provide many benefits in reducing clinical outcomes of type 2 diabetes. However, more preclinical and randomized clinical studies are needed to support our results.

## AUTHOR CONTRIBUTIONS

**Emrah Yazici:** Data curation (equal); formal analysis (equal); methodology (equal). **Emre Şahin:** Data curation (equal); formal analysis (equal). **Nurhan Sahin:** Methodology (equal). **Mehmet Tuzcu:** Formal analysis (equal). **Kazim Sahin:** Conceptualization (equal); writing – original draft (equal); writing – review and editing (equal). **Cemal Orhan:** Conceptualization (equal); data curation (equal); formal analysis (equal); investigation (equal); methodology (equal); writing – original draft (equal); writing – review and editing (equal).

## FUNDING INFORMATION

This project was funded by Firat University (FUBAP‐VF‐1720, Elazig, Turkey) and the Turkish Academy of Sciences (TUBA, KS) in part. The funders were not involved in the project design, collection, analysis, and interpretation of data, the writing of this article, or the decision to submit it for publication.

## CONFLICT OF INTEREST STATEMENT

The authors declare that they do not have any conflict of interest.

## ETHICS STATEMENT

The experimental procedures were approved by the Animal Experiments Local Ethics Committee of Firat University (protocol number: 2017/89‐169).

## Data Availability

The data that support the findings of this study are available on request from the corresponding author.
